# ROS and NO Regulation by Melatonin Under Abiotic Stress in Plants

**DOI:** 10.3390/antiox9111078

**Published:** 2020-11-03

**Authors:** Miriam Pardo-Hernández, Maria López-Delacalle, Rosa M. Rivero

**Affiliations:** Department of Plant Nutrition, Center of Edaphology and Applied Biology of Segura (CEBAS-CSIC), Campus Universitario Espinardo, Espinardo, 30100 Murcia, Spain; mpardo@cebas.csic.es (M.P.-H.); mlopez@cebas.csic.es (M.L.-D.)

**Keywords:** melatonin, ROS, NO, post-translational modifications (PMTs), abiotic stress, drought, salinity, high temperature, high light, waterlogging, abiotic stress combination

## Abstract

Abiotic stress in plants is an increasingly common problem in agriculture, and thus, studies on plant treatments with specific compounds that may help to mitigate these effects have increased in recent years. Melatonin (MET) application and its role in mitigating the negative effects of abiotic stress in plants have become important in the last few years. MET, a derivative of tryptophan, is an important plant-related response molecule involved in the growth, development, and reproduction of plants, and the induction of different stress factors. In addition, MET plays a protective role against different abiotic stresses such as salinity, high/low temperature, high light, waterlogging, nutrient deficiency and stress combination by regulating both the enzymatic and non-enzymatic antioxidant defense systems. Moreover, MET interacts with many signaling molecules, such as reactive oxygen species (ROS) and nitric oxide (NO), and participates in a wide variety of physiological reactions. It is well known that NO produces S-nitrosylation and NO_2_-Tyr of important antioxidant-related proteins, with this being an important mechanism for maintaining the antioxidant capacity of the AsA/GSH cycle under nitro-oxidative conditions, as extensively reviewed here under different abiotic stress conditions. Lastly, in this review, we show the coordinated actions between NO and MET as a long-range signaling molecule, regulating many responses in plants, including plant growth and abiotic stress tolerance. Despite all the knowledge acquired over the years, there is still more to know about how MET and NO act on the tolerance of plants to abiotic stresses.

## 1. Introduction

Abiotic or biotic stresses, such as drought, heat, cold, salinity, pathogen attack and high light, either individual or combined, negatively affect plant growth, reproduction and survival, which will limit agricultural crop productivity and yield [1]. Climate change events are predicted to increase the negative impact of environmental stress factors on plant production in many regions of the world in the coming years [2], with the unavoidable question of how humanity will cope with the world’s demand for food. Along with this, according to the Food and Agriculture Organization of the United Nations (FAO) (2017), the world population will increase to 9700 billion people by 2050 and the agricultural systems will therefore have to produce almost 50% more food, fodder and biofuels than in 2012. It has been shown that plant productivity is strongly affected when stresses act in combination [3]. Thus, the heat waves combined with a serious drought episode that affected Europe in 2003 led to a 30% reduction of the total European agricultural production [4]. Plants respond to environmental stresses through a variety of biochemical and molecular mechanisms, including the selective absorption and exclusion of ions, the compartmentalization of ions into the central vacuole, the synthesis and accumulation of organic solutes in the cytoplasm, the changes in membrane composition, and the alteration of plant hormone levels [3,5,6,7]. In this sense, molecules such as phytohormones, nitric oxide (NO), hydrogen sulfide and calcium (Ca^2+^) are directly involved in the plant’s response to environmental stresses [8,9]. Recently, numerous studies have shown that a new and recent plant molecule, melatonin (MET), also plays an important role in the response of plants to environmental stress, and its exogenous application has been shown to have a positive effect on the reduction of the impact of stress in plants [1,10], being cataloged as a phytohormone for its functions in plants [11].

## 2. Melatonin as an Elicitor of Plant Stress Response

MET (N-acetyl-5-methoxy-tryptamine) was first discovered in the bovine pineal gland in 1958 by Lerner et al. [12] and it was not discovered in higher plants until 1995, simultaneously by Dubbels et al. [13] and Hattori et al. [14]. MET has an indole ring structure, a low molecular weight, and is an evolutionarily conserved pleiotropic molecule that exists ubiquitously in living organisms. MET has an amphiphilic or amphipathic molecular character, which allows the molecule to easily pass through the cell membrane and move into the cytosol, the nucleus, and/or the mitochondria [15,16]. In plants, 2-hydroxymelatonin (2-OHMET) is the most abundant MET derivative, and its intracellular concentration is about two orders of magnitude higher than that of MET [17]. Thus, it is thought that this MET derivative may be more efficient in the induction of plant stress tolerance than MET [18].

**Figure 1:**
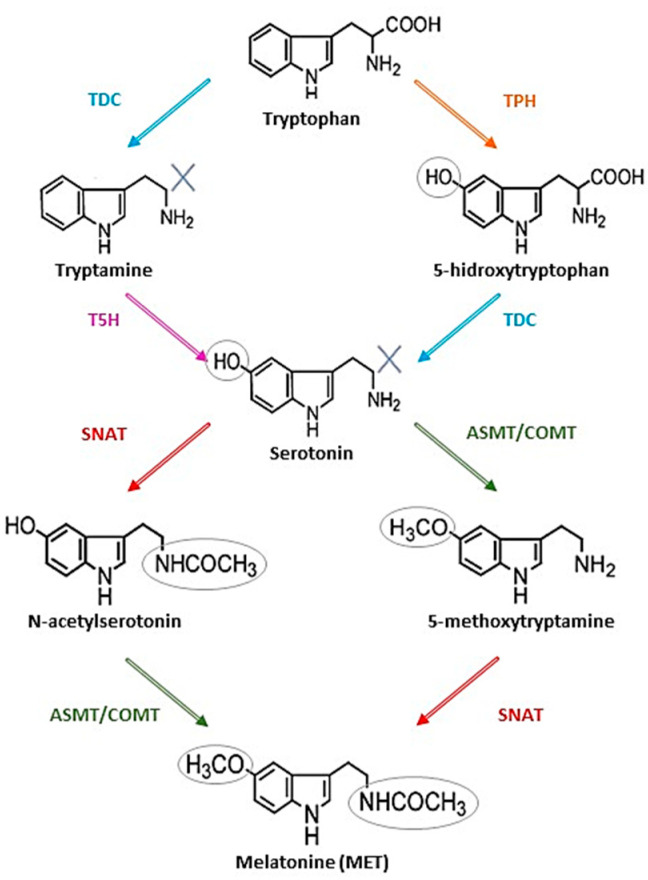
The four possible routes of melatonin (MET) biosynthesis. The enzymes that participate in the synthesis are: tryptophan decarboxylase (TDC), tryptophan hydroxylase (TPH), tryptamine 5-hydroxylase (T5H); serotonin N-acetyltransferase (SNAT); N-acetylserotonin methyltransferase (ASMT), and caffeic acid O-methyltransferase (COMT).

Starting with tryptophan, MET biosynthesis includes four enzymatic steps in all organisms; however, this varies among species [19]. In plants, tryptophan is first decarboxylated to tryptamine, which is then hydroxylated to serotonin, or the tryptophan is first hydroxylated to 5-hidroxytryptophan, which is then decarboxylated to serotonin. Serotonin is either acetylated to N-acetylserotonin or it is methylated to form 5-methoxytryptamine; these products are either methylated or acetylated, respectively, to produce MET [20] (Figure 1).

The content of MET in plants commonly differs between cultivars, species, growth and developmental periods, tissue types, and even in repetitions from a single experiment, and MET can be found in almost all the plant organs, from leaves to pistils [15,21]. Mitochondria and chloroplast are considered major sites of MET synthesis, although it can also be synthesized in the cytosol. The reactive oxygen species (ROS) are abundantly generated in mitochondria and chloroplast, and MET plays an important role in protection against free radicals in these organelles [20,22]. Especially in plants, MET is maintained at a relatively constant level under normal conditions, just as with ROS, and it is therefore believed that MET may mainly act as a regulator of ROS levels. Nevertheless, its synthesis and accumulation can be greatly and rapidly activated and upregulated in response to unfavorable conditions such as cold, heat, salt, drought, oxidative and nutrient stress, and bacterial infection [23,24]. On the other hand, it has been shown that under special circumstances, high levels of MET may be toxic to plants and may induce growth inhibition [25].

MET has been described as having many and important functions in plants. Several studies have reported that MET could be considered a growth regulator, as it plays a role in specific physiological processes in plants. MET regulates the growth of leaves, shoots and explants, and plays a role in leaf senescence. MET can also regulate plant vegetative growth processes such as rooting, leaf aging, photosynthetic yield, and biomass yield, and it plays a potential regulatory role in flowering processes and the formation and maturation of fruit and seeds [1,26,27]. Lastly, the evidence of the importance of MET in phytoremediation has been increasing [28]. But perhaps the most important function described for MET in living organisms is related to its role in non-receptor-mediated activities, such as scavenging of ROS and reactive nitrogen species (RNS) and improving the cell’s antioxidant capacity, preventing cells, tissues, and organisms from suffering oxidative stress [15]. In this sense, MET modulates and quickly upregulates the activity of different antioxidant enzymes and stress tolerance-related genes, and activates downstream signaling transduction pathways when the plant is exposed to abiotic or biotic stress or the perception of MET [29,30]. The perception of MET by receptors could directly modulate expression of stress-responsive genes, or indirectly by MET signaling components or secondary messengers such as ROS/RNS. For example, MET upregulates the C_2_H_2_-type zinc finger transcription factors *ZAT*s, which act as upstream regulators of the CBFs/DREBs pathway during cold stress responses in plants [31]. MET also regulates salinity through the *SOS* pathway [32], and the abscisic acid (ABA) signaling transduction pathway is modulated by MET for regulating the plant’s drought-stress response [33]. In 2018, Dr. Chen’s group first detected and characterized the phytoMET receptor PMTR1. PMTR1-phytoMET binding triggers the dissociation of G_γb_ and G_α_, which activates NADPH oxidase-dependent H_2_O_2_ production, enhancing Ca^2+^ influx and promoting K^+^ efflux, all of which results in stomatal closure [11,34]. In addition, MET can interact with unknown receptors in the activation of H_2_O_2_/NO signaling pathways, and further improve plant stress tolerance by regulating a variety of antioxidant enzymes, thus alleviating photosynthesis inhibition and modulating the activity of various transcription factors [35].

## 3. ROS-Plant Mediated Stress Response and Its Relationship with MET

For oxygen-dependent lifeforms, the formation of ROS and its coexistence within the cells are an unavoidable part of oxygen-evolving organisms. ROS are permanently generated in most of the plant cell’s organelles (chloroplast, mitochondria, peroxisomes and cytosol) and the apoplast. Due to the harmful effects of ROS, cells have had to evolve different and effective detoxification mechanisms, through a network that coordinates antioxidant enzymes and non-enzymatic compounds. Well-known antioxidant enzymes include superoxide dismutase (SOD), catalase (CAT), peroxidase (POD), ascorbate peroxidase (APX) and glutathione peroxidase (GPX), while representative non-enzymatic compounds include glutathione (GSH), ascorbic acid (AsA), proline (Pro), carotenoids, α- tocopherols, etc. Moreover, a plethora of metabolites, including carbohydrates and amino acids, are generally accumulated to protect plants against abiotic stress [36,37,38]. Other antioxidant molecules that improve plant tolerance in plant tissues are different phenolic [39] and flavonoid compounds [40], although in the present review we will only focus on the increasingly recognized and important antioxidant, MET.

**Figure 2:**
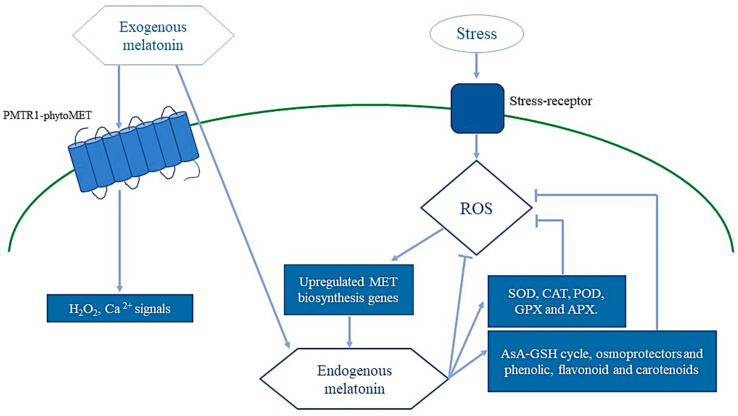
Interaction between melatonin (MET) and reactive oxygen species (ROS). ROS upregulates MET biosynthesis genes and enhances MET endogenous levels. MET can act as a ROS scavenger and controlled ROS levels through the melatonin-mediated induction of redox enzymes, such as SOD, CAT, POD, GPX and APX, and representative antioxidant representative non-enzymatic antioxidant compounds such as GSH and AsA (AsA-GSH cycle), osmoprotectants, and phenolic, flavonoid and carotenoid compounds. Similarly, the exogenous MET interacts with its receptor (CAND2/PMTR1), which appears to be melatonin-induced, resulting in the activation of responses against stressors are feed into a feedback mechanism with different regulating elements that belong to the redox network, such as ROS and RNS [42].

ROS play a fundamental role as signaling molecules in the regulation of numerous biological processes such as growth, development and responses to biotic and/or abiotic stress in plants. Stress responses in plants are mediated by a temporal–spatial coordination between ROS and other signals that rely on the production of stress-specific chemicals, compounds and hormones [41]. Higher ROS levels in plants occur when the plants are under stress. The ROS, mainly ^·^OH, O_2_^−^^.^, H_2_O_2,_ act as signaling plant molecules and activate different mechanisms related to stress tolerance. Recently it was shown that one of these ROS-regulated molecules is MET. Interestingly, Arnao and Hernadez-Ruiz [42] showed that ROS are able to upregulate the MET biosynthesis pathway genes and, therefore, enhance the plant’s endogenous levels of MET [43]. In turn, endogenous MET can act as an effective ROS scavenger [44,45]. Thus, MET controls ROS levels in two different ways: through its chemical interaction with ROS which leads to their inactivation [42]; or by the MET-mediated induction of the redox enzymes that detoxify ROS, such as SOD, CAT, POD, GPX and APX [46]. On the other hand, MET also induces the accumulation of some representative non-enzymatic antioxidant compounds such as GSH and AsA [47,48,49,50,51,52,53,54], phenolic compounds [39], flavonoid via the NO-dependent pathway [40] and carotenoids [33,55]. Similarly, the exogenous application of MET was shown to interact with its plant receptor (CAND2/PMTR1), which appears to be MET-induced, resulting in the activation of responses against stressors [43] (Figure 2). In brief, the actions of METgo into a feedback mechanism with different regulated elements belong to the redox network, such as ROS and RNS [42].

## 4. NO-Plant Mediated Stress Response and Its Relationship with MET

**Figure 3:**
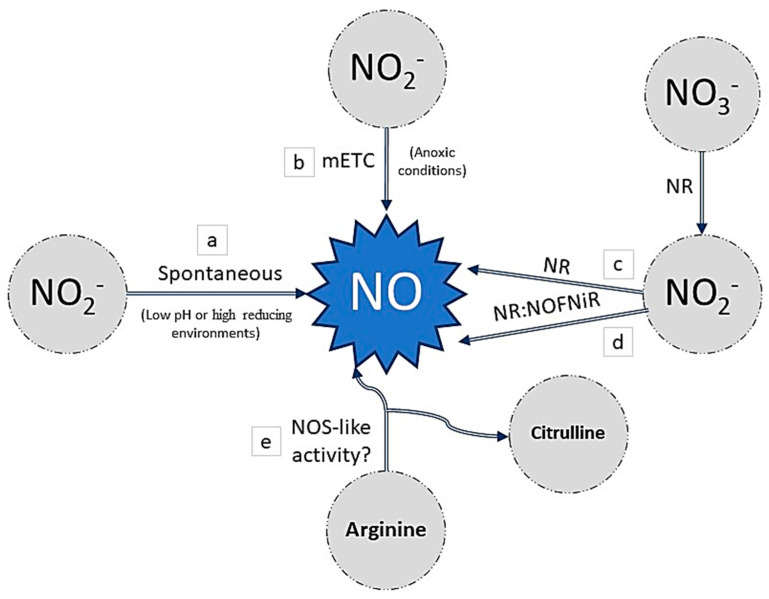
Nitric oxide (NO) synthesis in plants: (**a**) the non-enzymatically mechanism, under low pH or high reducing environments. (**b**) NO can be produced from nitrite through the action of the mitochondrial electron transport chain (mETC). (**c**) Nitrate reductase (NR) is responsible for the reduction of nitrate to nitrite using and then producing NO by Ni-NR activity. (**d**) NR can interact with the partner protein NOFNiR (nitric oxide-forming nitrite reductase) to produce NO from nitrite. (**e**) Arginine-dependent pathway in plants forms NO by NOS (nitric oxide synthase)-like activity.

Nitric oxide (NO) is another key signaling molecule in plant physiology. The most described and evidenced NO production route involves the reduction of nitrite to NO via different non-enzymatic and/or enzymatic mechanisms in plants [56] (Figure 3). When high concentrations of nitrate are present in a low pH or high reducing environments, the reduction of nitrites to NO could efficiently and rapidly occur non-enzymatically [57] (Figure 3a). On the other hand, several proteins have been described as being able to catalyze the production of NO from nitrites, and one of them is nitrate reductase (NR). NR is a multifunctional cytoplasmic enzyme that is responsible for the reduction of nitrate to nitrite using NADH as an electron donor, and it is the first limiting step of nitrate assimilation [58]. This enzyme also has a nitrite:NO reductase activity (Ni-NR activity), from which NO is produced. However, this second reaction represents only 1% of the nitrate-reducing capacity of NR in normal conditions. Nevertheless, the reaction could be promoted by specific conditions such as anoxic conditions [59,60,61] (Figure 3c). Furthermore, in *Arabidopsis thaliana*, it has been shown that NR can interact with the partner protein NOFNiR (nitric oxide-forming nitrite reductase) to produce NO from nitrite. In contrast to the Ni-NR activity, the NO-producing activity occurs under normoxia and is not inhibited by nitrate [62] (Figure 3d). Moreover, NO can be produced from nitrite through the action of the mitochondrial electron transport chain (mETC) in plants, which requires anaerobic conditions and depends on the availability of nitrite [63,64] (Figure 3b). Apart from these reductive routes, several lines of evidence demonstrate the existence of an oxidative route for NO production in an arginine-dependent pathway in plants [56]. At present, it is evident that embryophyte transcriptomes do not have canonical nitric oxide synthases (NOSs). However, several pieces of evidence reported in various articles are in favor of the existence of NOS-like activity. This activity is dependent on arginine, or at least the arginine metabolic pathways (Figure 3e). The identification and description of the proteins and substrate/cofactors involved are necessary for a better understanding of NO formation in plants [56,65].

In plant cells, NO plays many important roles in the regulation of the cellular redox balance through post-translational modifications (PTMs) and/or through its binding to the prosthetic hemo group of a few antioxidant enzymes. The PTMs induced by NO include S-nitrosylation, tyrosine nitration and metal nitrosylation, although the first two are the most important at a physiological level [66]. S-nitrosylation is the covalent binding of NO to the thiol group of cysteines. In many antioxidant target proteins, S-nitrosylation leads to the regulation of the protein’s function during stress [67,68]. Likewise, protein tyrosine nitration (NO_2_-Tyr) consists of the addition of a nitro (—NO_2_) group to one of the two equivalent ortho carbons of the aromatic ring of tyrosine residues [69]. This NO_2_-Tyr is considered a selective process, as under physiological conditions, the nitrotyrosine concentration is very low (from 1–5 NO_2_-Tyr among 10,000 molecules of tyrosine) [70]. In the same manner as S-nitrosylation, NO_2_-Tyr is able to alter protein functions through a gain, no change or loss of function, with the latter being the most common in plants [66,71]. Numerous data obtained show an interrelationship between S-nitrosylation and NO_2_-Tyr in the regulation of the activity of some antioxidant proteins, being an important mechanism for maintaining the antioxidant capacity of the AsA/GSH (ascorbic acid/glutathione) cycle under nitro-oxidative conditions [72,73]. NO also regulates other important proteins, thereby inducing other cellular processes. NO functions as a Ca^2+^-mobilizing messenger by promoting the rise in cytosolic Ca^2+^ concentrations. By increasing cytosolic Ca^2+^ concentration, NO modulates the activity of protein kinases and Ca^2+^-sensitive channels, which may be involved in the signaling cascade that leads to the expression of defense-related genes (adaptive response to biotic and abiotic stresses), stomatal closure, or adventitious root formation and germination. These processes involve cyclic adenosine diphosphate (cADP) ribose, cyclic guanosine monophosphate (cGMP), and protein kinases [74].

Additionally, NO can be used commercially in postharvest fruit programs for the improvement of antioxidant and defense systems, the inhibition of ethylene biosynthesis, the activation of the C-repeat-binding factors (CBFs) pathway, and the regulation of sugar and energy metabolisms [75]. The signaling role of NO in plants has also been reported to modulate plant growth in normal and stress conditions [76]. Treatment with exogenous NO prevents damage from stress, promotes disease resistance, delays fruit ripening and enriches the nutritional quality of fruits [77]. In normal growth conditions, Wen et al. (2016) have suggested that elevated NO levels resulting from the upregulated expression of NR, the down-regulation of S-nitrosoglutathionereductase (GSNOR), and auxin signaling were involved in the MET-induced adventitious root formation in tomato (*Solanum lycopersicum* L.) plants [78].

**Figure 4:**
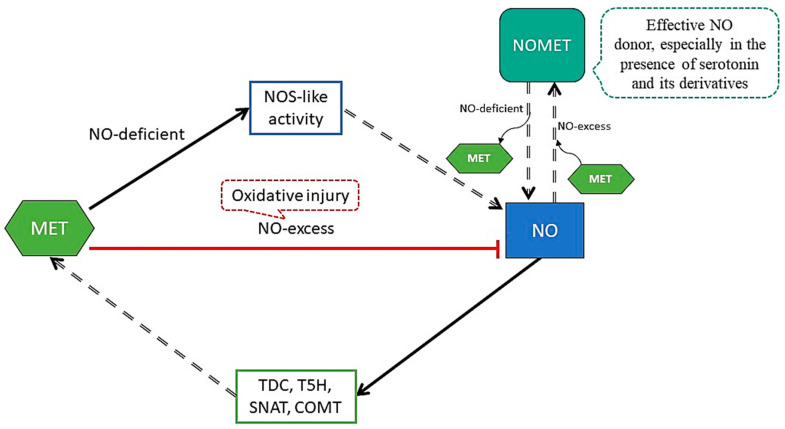
Interaction between melatonin (MET) and nitric oxide (NO). MET promotes the accumulation of NO by increasing the activity of NOS (nitric oxide synthase)-like since MET up-regulates the expression of related genes. MET scavenges excess NO, as it produces oxidative injury (red arrow). In the presence of oxygen, MET can be easily converted to N-Nitrosomelatonin (NOMET) by NO nitrosation under different pH conditions. As well, NOMET is an effective NO donor in cell cultures under the presence of serotonin and its derivatives. On the other hand, through a cyclic guanosine monophosphate (cGMP)-dependent pathway, NO induces the expression of TDC, T5H, SNAT and COMT (genes of the enzymes of the MET biosynthesis pathway) to increase MET levels.

MET acts together with NO in plant growth and mediates plant abiotic stress tolerance through the improvement of the antioxidant system [79] (Figure 4).

Just as MET, NO can directly scavenge free radicals and reduce oxidative damage in a receptor-independent manner under external stimuli and developmental signals [80]. MET can induce NO production or scavenge excess NO, and can promote the accumulation of NO by increasing the activity of NOS-like protein (arginine metabolic pathway), as MET up-regulates the expression of related genes [81,82]. In the presence of oxygen, MET can be easily converted to N-Nitrosomelatonin (NOMET) by NO nitrosation under different pH conditions. However, under the presence of serotonin and its derivatives, NOMET is an effective NO donor in cell cultures [83,84]. On the other hand, through a cGMP-dependent pathway, NO induces the expression of TDC, T5H, SNAT and COMT (genes of the enzymes from the MET biosynthesis pathway) to increase the levels of MET [79]. The interaction between NO and MET shows a certain degree of intricacy, as they interact independently and through multiple signaling pathways [85].

Taking into consideration the new progress in MET studies in recent years, the activity of MET in plants has been comprehensively and intensely explored. Here we compile the updated research works conducted on the relationship of MET with the stress tolerance mechanisms of plants, and the elucidated interaction between MET, ROS and NO in the induction of this tolerance under different environmental stresses.

## 5. MET under Environmental Stress and the Roles of ROS and RNS

Plants live in constantly changing environments that are often unfavorable or stressful for their growth and development. These adverse environmental conditions include biotic and abiotic stresses. Some of the most important abiotic stresses are salinity, heat/cold stress, drought, waterlogging, high light, and nutrient deficiency, which affect and ultimately determine the geographical distribution of plants in nature, limit plant productivity in agriculture and threaten food security. Moreover, the adverse effects of these abiotic stresses are aggravated by the inevitable effects of climate change, which has been predicted to result in the increased frequency of extreme weather [86,87]. Thus, improving plant stress resistance is critical for ensuring agricultural productivity and also for environmental sustainability, as it has also been shown that crops with poor stress resistance consume too much water and fertilizers [87].

First, salt stress increases inter- and intracellular accumulation of sodium ions (Na^+^), inhibits protein synthesis and enzyme activities, damages cellular organelles and uncouples photosynthesis and respiration. Next, salinity decreases nutrient uptake and/or transport to the shoot, resulting in a nutrient imbalance; and lastly, salinity decreases soil osmotic potentials which impedes water uptake by roots [88,89]. Heat stress (5 °C above the optimal growth temperature) is commonly related to three types of damage: oxidative stress caused by ROS; dicarbonyl stress induced by methylglyoxal, which rapidly reacts with biomacromolecules (proteins, lipids and nucleic acids); dehydration or desiccation of cells due to water deficit caused by osmotic stress; and loss of biomembrane integrity [90,91,92,93]. In addition, heat stress boosts the endogenous levels of putrescine, spermidine and spermine, and increases the content of PAs, indicating a higher metabolic gene expression [51]. In plants, cold stress exerts various effects, resulting in physiological, biochemical and molecular changes [94,95]. Cold stress directly affects the photosynthetic machinery, principally by inducing photoinhibition at both PSI and PSII levels [96]. The main deleterious effects of drought stress are the reduction in relative water content, reduction in water potential of the leaf, loss of turgor and cell enlargement reduction, which further results in the decrease of photosynthetic pigments, disturbance of different metabolic processes, and lastly, plant death [97,98]. Light is essential for photosynthesis but high light plays a role as an environmental stressor which elicits ROS production in plants, resulting in extensive photooxidative damage [99]. Specifically, natural levels of UV-B (280–315 nm wavelength) play important roles in the regulation of plant growth and development. Nevertheless, elevated UV-B doses can induce deleterious effects, including disruption of the integrity and function of important macromolecules (DNA, lipids and proteins), oxidative damage and photosynthesis deficiency [100]. Extended exposure to UV-B may also negatively affect growth and productivity and may produce photomorphogenic effects on plants [101,102]. In general, abiotic stresses induce a common oxidative burst in plants. This consists of a general disruption of redox homeostasis, causing an imbalance on the production and scavenging of ROS, which reduces photosynthesis and induces stomatal closure, and alters the activities of enzymes [41].

Plant responses to abiotic stress are dynamic and complex. Different experiments have revealed the kinetics of stress responses through the identification of multiple response phases that involve core sets of genes and condition-dependent changes. To decrease damage due to stress, plants have evolved different pathways. The increase of ROS and RNS within the cells is one of the first responses to stress. The formation and accumulation of ROS and RNS is especially important in plants, as they can cause electron leakage (EL), lipid peroxidation and subsequent membrane damage, as well as damage to nucleic acids and proteins [103].

### General Roles of MET in Abiotic Stress Tolerance

**Figure 5:**
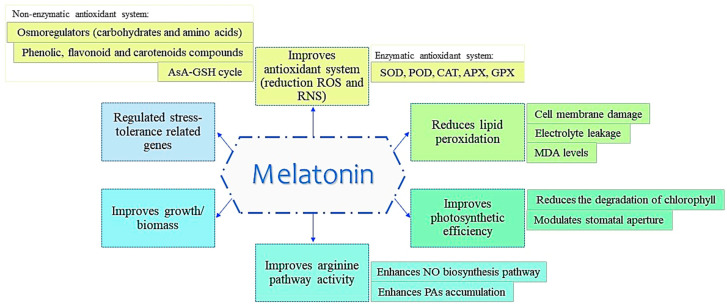
Common MET functions in abiotic stress tolerance.

In general, the mechanisms that involve MET in the tolerance to abiotic stress are similar (Figure 5), although some MET mechanisms are specific to a certain type of stress, which will be specifically described below. Table 1 shows the different effects of MET in response to abiotic stress, depending of the type of abiotic stress and the plant species. Principally, MET supplementation is associated with a decrease in ROS levels and redox homeostasis due to the enhanced scavenging activity or expression of the antioxidant enzymes, including SOD, CAT, POD, APX and GPX, under salinity in tomato, sunflower, alfalfa (*Medicago sativa* L.), *Malus hupehensis* Rehd., watermelon, *Leymus chinensis* and strawberry plants [48,104,105,106,107,108,109], high temperature in tomato, maize (*Zea mays* L.), kiwifruit (*Actinidia deliciosa*) and tall fescue plants [50,51,52,53,110,111], cold in tomato, cucumber and *Camelia sinensis* L. plants [36,54,95,112], drought in *Carya cathayensis*, *Moringa oleifera* L., maize, *Medicago sativa* L., naked oad, soybean and cucumber plants [33,98,113,114,115,116,117,118], high light in *Malus hupehensis* plants [102], waterlogging in *Malus baccata* (Linn.) plants [119] and stress combination in tomato, rice and pepper (*Capsicum annuum* L.) plants and *Haematococcus pluvialis* [10,18,120,121]. Additionally, MET application improves representative non-enzymatic antioxidant compounds such as GSH and AsA (AsA-GSH cycle) under stress due to salinity in sunflower, tomato and sweet potato plants [47,48,49], high temperature in tomato, maize (*Zea mays* L.) and kiwifruit *(Actinidia deliciosa*) plants [50,51,52,53] and cold in cucumber plants [54]. For example, Cen et al. (2020) confirmed that MET performed its primary function as an antioxidant, positively improving the salt tolerance of alfalfa (*Medicago sativa* L. cv. Zhongmu No. 2) through ROS scavenging and the enhancement of the activities of antioxidant enzymes [105]. Lastly, Zahedi et al. (2020) indicated that the effects of MET application were associated with a boost in leaf antioxidant enzymes and ABA, and provided support for the hypothesis that the application of MET was a promising tool for mitigating salt stress in strawberry (*Fragaria* × *ananassa* Duch., cv. ‘Camarosa’). Current results show evidence of an interaction between MET and NO in sunflower (*Helianthus annuus* L. var. KBSH 54) seedlings grown under salinity stress, with a differential modulation of two SOD isoforms (Cu/Zn SOD and Mn SOD) [104]. Moreover, MET and NO differentially and coordinately ameliorate the salt stress effect by modulating glutathione reductase (GR) activity and GSH content in sunflower cotyledons (*H. annuus* L. cv. KBSH 53) seedlings [47]. MET was also found to be involved in the heat-tolerant response; for example, in kiwifruit (*Actinidia deliciosa*), it was observed that MET exerted a protective effect against heat-related damage via the increase of AsA levels and the activity of several antioxidant enzymes. These changes were shown to be accompanied by an increase in the activity of enzymes linked to the AsA-GSH cycle, such as APX, monodehydroascorbate reductase (MDHAR), dehydroascorbate reductase (DHAR), and GR. Furthermore, MET application increased the expression of 28 out of 31 glutathione S-transferase genes, which drove to the reduction of the oxidative stress generated under heat stress [53]. In tall fescue (*Festuca arundinacea* Schreb.), Alam et al. (2018) also found that MET effectively improved total protein and antioxidant enzyme activities under heat stress conditions, resulting in improved plant growth. In naked oat (*Avena nuda* L.), the effects of exogenous MET on the antioxidant capacity of the plants under drought stress has also been shown. The results showed that a MET pretreatment reduced the levels of H_2_O_2_ and O_2_^−^ and enhanced SOD, POD, CAT and APX activities [115].

Other non-enzymatic antioxidant molecules which increased in concentration after the addition of MET are phenolic compounds in canola plants (*Brassica napus* L.) [39], flavonoids via the NO-dependent pathway [40] and carotenoids compounds in *Carya cathayensis* and *Arabidopsis thaliana* plants [33,55]. Moreover, MET treatment enhances osmoregulation with molecules such as carbohydrates (trehalose) and amino acids (Pro), which are commonly accumulated to protect plants against abiotic stress such as high temperature in maize (*Zea mays* L.) and kiwifruit (*Actinidia deliciosa*) seedlings [52,53], drought in *Carya cathayensis* and maize seedlings [33,113] and stress combination (cold and drought) in rice plants [18].

In addition, MET application induces a higher arginine pathway activity, and therefore, a higher endogenous polyamines (PAs) accumulation (further increasing levels of PAs and upregulating transcript abundance, which coincided with the suppression of catabolic-related gene expression) under abiotic stress such as salinity in *Malus hupehensis* Rehd. [106], high temperature in tomato plants [51], cold in tomato plants [81], and waterlogging in alfalfa plants (*Medicago sativa* L.) [122]. In this sense, in alfalfa plants (*Medicago sativa* L.), an exogenous application of MET was also significantly involved in mitigating waterlogging stress [122]. The researchers observed that first, MET suppressed ethylene production through the downregulation of the ethylene biosynthesis-related genes and the mitigation of waterlogging-induced growth reduction, chlorosis, and premature senescence in plants. Subsequently, MET increased PA content by enhancing the activity and gene expressions of the PA metabolism enzymes. Thus, MET mitigated waterlogging stress through cross-talk with or by directly modulating the metabolic pathways of PAs and ethylene in alfalfa [122].

An interaction of MET with NO has been observed under salinity, high temperature, cold, drought, Fe deficiency and the combination of high light and N deficiency stresses. MET enhances the NO biosynthesis pathway through the regulation of endogenous NO content, NR and NO synthase-related activities (via the arginine pathway), and the expression of their related genes [51,81,114]. On the other hand, NO also upregulates MET, as NO regulates the MET synthetic enzymes [47] and modulates MET accumulation through the formation of NOMET [104]. MET is transported in the form of a metabolic signal (NOMET) from the roots, across the hypocotyl, until reaching the cotyledon cells within a time frame of 48 h after radical emergence, leading to a reduction in both oxidative and nitrosative stress in sunflower seedlings under salt stress. That is, NO plays a role as a positive modulator of MET accumulation in seedling cotyledons in a long-distance signaling response [104]. Additionally, MET and NO differentially and coordinately ameliorate the salt stress effect by modulating glutathione reductase (GR) activity and GSH content in sunflower cotyledons seedlings. MET content in these organs was also modulated by NO, which upregulated the activity of hydroxyindole-O-methyltransferase - a regulatory enzyme in MET biosynthesis in response to salt stress [47]. The inteconnection between MET and NO is not yet well-known, however, quite a few research studies are in agreement in that MET increases NO levels under abiotic stress [32,47,51,81,104,114,120,121,123].

Under abiotic stress, various plants respond with the formation of NO. In several plants, NO is a key signaling molecule needed for the regulation of responses such as photosynthesis, oxidative defense, osmolyte accumulation, gene expression and protein modifications [85]. Zhao et al. (2018) presented genetic and pharmacological evidence demonstrating the involvement of NO in MET signaling for salinity tolerance in rapeseed (*Brassica napus* L. zhongshuang 11). MET triggered a signaling cascade that resulted in the induction of NR and NO associated 1- (NOA1) dependent NO concentration. In rapeseed, it was demonstrated that the removal of NO did not alter endogenous MET content in roots supplemented with NaCl alone or together with MET; thus, NO was not responsible for MET production [123]. Zhao et al. (2018) also discovered that NaCl-induced S-nitrosylation was intensified by MET and sodium nitroprusside (SNP), although it decreased by the removal of NO [123]. More specifically, under alkaline-saline stress, it was observed that exogenous MET treatment elevated NO levels in tomato (*S. lycopersicum* L.) roots, increasing their tolerance to alkaline stress. The treatment with MET and NO reduced damage caused by alkaline stress through the reduction of Na^+^ accumulation, activated the expressions of genes from the defense response signal pathway, and improved the uptake of K^+^, antioxidant enzyme activity and AsA–GSH detoxification capacity [35]. In brief, pharmacological, molecular and genetic data have provided solid evidence that NO operates downstream of MET, enhancing salinity tolerance, which opens a new door for future research on the signaling mechanisms involved in plant stress tolerance. A research study with tomato (*S. lycopersicum* L. cv. Hezuo 903) seedlings demonstrated that heat stress-induced damage was suppressed by MET, which coordinated with the PA and NO biosynthesis pathways. MET improves the NO biosynthesis pathway (increasing endogenous NO content, NR and NO synthase-related activities, and the expression of their related genes). Therefore, the cross-talk that exists between MET, PAs and NO is related to the inhibition of heat stress effects [51]. In tomato (*Lycopersicon esculentum* cv. Izmir) fruits, the application of exogenous MET conferred chilling tolerance. MET treatment increased the arginine pathway activity in tomato fruits; therefore, a higher endogenous PA accumulation and higher endogenous NO accumulation arising from higher NOS gene expression and enzyme activity may be responsible for maintaining safe membrane integrity by lowering EL and malondialdehyde (MDA) accumulation during chilling stress [81]. Other results revealed that the rhizospheric application of MET remarkably enhanced the drought tolerance of alfalfa (*Medicago sativa* L.) plants through nitro-oxidative homeostasis via the regulation of reactive oxygen (SOD, GR, CAT, APX) and nitrogen species (NR, NADH dehydrogenase) metabolic enzymes at the enzymatic and/or transcript level. MET pre-treatment was correlated with the subsequent significant down-regulation of NR transcript levels, which is the key biosynthetic enzyme for NO generation in plants [114].

Currently, there are no studies that link MET with NO in the response to high light stress, waterlogging and nutrient deficiency. However, different studies have shown that NO is involved in the tolerance to high light stress [124]. Kim et al. (2010) demonstrated that NO was involved in the high light tolerance of maize (*Zea mays* L.) leaves of seedlings. The NO donor permitted the survival of more green leaf tissue than in non-treated controls under UV-B stress. Moreover, NO-treated seedlings had an increased concentration of flavonoids and anthocyanins, UV-B absorbing compounds and MDA. Lastly, the NO donor under UV-B stress increased the CAT and APX activities to a greater degree [40]. In the same way, UV-B strongly induced NO production, which protected the green alga *Chlorella pyrenoidosa* against UV-B-induced oxidative damage [125]. On the other hand, it has been observed that in the response to stress due to high light, NO interacts with other molecules. For example, the interplay between NO and inositol signaling can be involved in the mediation of UV-B-initiated oxidative stress in *A. thaliana* cells [126] or the causal and interdependent relationship between NO and H_2_O_2_ induces stomatal closure in broad bean (*Vicia faba* L.) under UV-B [127]. Similarly, it is considered that salicylic acid, SNP, and especially their combination, could alleviate UV-B stress in dwarf polish wheat (*Triticum polonicum* L.) [128]. Ultimately, Cassia et al. (2019) showed that UV-B perception triggered an increase in ABA concentration, which increased H_2_O_2_ and induced NO [129]. In addition, in soybeans (*Glycine max* L.), Khan et al. (2019) observed that the application of a NO donor could mitigate the negative effects of short-term flooding stress through the reduction of oxidative stress [130]. Likewise, in two varieties of wheat (*Triticum aestivum* L. cv. Dogankent and *T. aestivum* cv. Ducula-4). the application of waterlogging and waterlogging + NO increased antioxidant enzyme activity, as well as the expression of the genes that were studied, for example, Myb2, PDPK, and SST1, specifically during the early hours of treatment, although this was higher in the wild type + NO treatment [131]. On the other hand, Chen et al. (2016) have provided support to the hypothesis that NO increases waterlogging tolerance by increased adventitious root formation and endogenous NO production via the upregulation of NOS activity in *Suaeda salsa* L. [132]. Lastly, Copolovici and Niinemets (2010) demonstrated that the tolerance to flood stress was proportionally related to the amount of NO emitted during longer-term conditions under this stress [133]. Additionally, the rate of NO synthesis or NO degradation was normally affected by nutrient imbalances. In turn, changes in the level of NO modified plant morphology and/or regulated nutrient homeostasis, through its interaction with reactive oxygen species and phytohormones, and through the post-translational modification of proteins [134]. At present, studies on the relationship of MET with NO in response to high light stress, waterlogging and nutrient deficiency, have not been found. However, as with most stresses, this relationship must exist in the response to this particular stress.

Perhaps one of the most important functions of MET in plants is the improvement of the photosynthetic efficiency under most abiotic stresses such as salinity [48,107,108,135,136], high temperature [137], cold [36,54,95], drought [33,98,113,118], high light [102,138], waterlogging [119,122,139,140], S deficiency [141] and stress combination [10]. Zhou et al. (2016) verified that MET improves the photosynthetic activities of tomato (*Solanum lycopersicum* cv. Jin Peng Yi Hao) seedlings under salinity stress. These authors demonstrated that MET controlled ROS levels and prevented the damage caused by the high cellular concentration of ROS caused by salinity stress, thereby promoting the recovery of the photosynthetic electron transport chain (PET) and D1 protein synthesis. In this sense, MET enhanced the photosynthetic activities and the repair of the light reaction-related proteins under salinity stress [136]. A pretreatment with MET of watermelon seedlings (*Citrullus lanatus* L. cv. 04-1-2) alleviated NaCl induced in roots through the inhibition of stomatal closure and thus, through the protection of the photosynthesis apparatus. It was also shown that MET improved light energy absorption, PET in PSII and redox homeostasis coupled with the enhanced activities of antioxidant enzymes [107]. Another more detailed study in grafted Chinese hickory (*Carya cathayensis*) plants revealed that exogenously applied MET successfully recovered the growth of plants and improved photosynthetic efficiency under dought stress [33]. The results of this work showed that the exogenously applied MET resulted in enhanced ROS scavenging and the accumulation of compatible solutes such as total soluble sugars and Pro. Moreover, the analyses using metabolomics demonstrated that drought-stressed plants treated with MET showed differentially regulated key metabolic pathways such as the phenylpropanoid, Chl and carotenoid biosynthesis pathways, carbon fixation, and sugar metabolism [33]. Moreover, exogenous MET reduced the negative effect of excess light by increasing the efficiency of the photosystems and rearranging the expression of chloroplast- and nuclear-encoded genes (housekeeping genes involved in the maintenance of transcriptional activity and the functional state of chloroplasts) in detached *A. thaliana* leaves [138].

As previously mentioned, MET is considered to be a growth regulator, as it can regulate plant vegetative growth processes such as rooting, leaf aging, photosynthetic yield and biomass yield, and it plays a potential regulatory role in flowering processes and the formation and maturation of fruit and seeds [1,26,27]. As such, MET is considered a phytohormone and thus is able to crosstalk with other plants hormones (zeatin, gibberellin A14, 24-epibrassinolide, jasmonic acid, and ABA) to regulate these physiological processes [33]. Thus, Fu et al. (2017) discovered that the exogenous application of MET enhanced cold tolerance via the induction of endogenous MET production, which may serve as a secondary messenger, activating downstream cold-responsive genes. Their results indicated that both ABA-dependent and ABA-independent pathways may have contributed to MET-induced cold tolerance in *Elymus nutans* [142]. Moreover, MET suppressed ethylene production through the downregulation of the ethylene biosynthesis-related genes and the mitigation of waterlogging-induced growth reduction, chlorosis, and premature senescence in plants [122]. Subsequently, MET increased PA content by enhancing the activity and gene expressions of the PA metabolism enzymes. Thus, MET mitigated waterlogging stress through cross-talk with or by directly modulating the metabolic pathways of the PAs and ethylene in alfalfa [122]. Nevertheless, more studies are needed to elucidate the interconnection between MET and other signaling molecules in response to abiotic stresses.

Finally, various studies have confirmed that MET significantly upregulated stress tolerance-related genes under salinity stress (membrane Na^+^/H+ antiporter (*SlSOS1*), vacuolar Na^+^/H+ exchanger (*SlNHX1*), and Na^+^ transporter (*SlHKT1,1* and *SlHKT1,2*) [32], high temperature stress (including FaHSFA3, FaAWPM and FaCYTC2) [110], low temperature stress (COR15a, a cold responsive gene, CAMTA1, a transcription factor involved in freezing and drought-stress tolerance, and transcription activators of ROS-related antioxidant genes ZAT10 and ZAT12) [31,142], drought stress (mitogen-activated protein kinases (MAPKs) Asmap1 and Aspk11, and the transcription factor genes such as WRKY1, DREB2, and MYB) [115] and S deficiency stress (genes encoding the enzymes involved in S transport and metabolism) [141]. Similarly, MET can also downregulate genes such as those found in waterlogging stress (downregulation of the ethylene biosynthesis-related genes) [122]. Similarly, in rice seeds (*Oryza sativa* cv. Dongjin), 2-OHMET alleviated the combined effects of cold and drought stresses via the actions of multiple TFs, including Myb4 and AP37, whereas no tolerance was observed in seedlings treated with either MET or water (control). The tolerance phenotype was associated with the induction of several transporter proteins, including a proton transporter (UCP1), a potassium transporter (HKT1) and a water channel protein (PIP2;1) [18].

Some more specific studies about stress tolerance in plants induced by MET have demonstrated that MET is able to regulate more specific mechanisms depending on the abiotic stress. Yang et al. (2020) provided new insights on the beneficial effects of exogenous MET on saline-alkaline stress tolerance in mycorrhizal *Leymus chinensis* (Trin.) through the regulation of the antioxidant systems, the protection of photosynthetic activity and the promotion of associated arbuscular mycorrhizal fungal growth, without changing soil salinity and alkalinity [108]. On other hand, high temperature also induced MET biosynthesis as a result of the accumulation of heat-induced Heat-sock factors (Hsfs) and heat shock proteins (HSPs) in tomato plants (*S. lycopersicum* L. cv. Micro-Tom). The accumulation of Hsfs stimulated the transcription of SNAT, an enzyme involved in MET biosynthesis. Simultaneously, HSP40 interacted with SNAT, escaping natural SNAT degradation which occurs under heat stress [143]. Other innovative research studies have shown that the high temperature-induced pollen abortion decreased with the MET treatment, as it provided protection against the degradation of organelles by enhancing the expression of HSPs genes to refold unfolded proteins, and the expression of autophagy-related genes and formation of autophagosomes to degrade denatured proteins [111].

Moreover, specifically under Fe deficiency in plants, exogenous MET increased the soluble Fe content of shoots and roots, and decreased the levels of root cell wall Fe bound to pectin and hemicellulose, allowing for the remobilization of cell wall Fe and the alleviation of Fe deficiency-induced chlorosis. Additionally, Fe deficiency quickly induced MET biosynthesis, acting synergistically with exogenous treatments [144]. Lastly, in pepper (*Capsicum annuum* L. cv Semerkand) plants, the tolerance induced by MET to Fe deficiency or salinity stress, applied individually, was shown to involve the downstream signal crosstalk between NO and H_2_S. However, MET was not very effective when Fe deficiency and salinity stress were applied together [121]. Consequently, more studies are necessary to elucidate a possible role of MET and NO in the tolerance mechanisms induced by field conditions.

## 6. Conclusions and Future Perpectives

Melatonin (MET) seems to play a relevant role in alleviating abiotic stress directly through the scavenging of ROS and RNS, and indirectly through enhancing antioxidant activities and photosynthetic capacity, regulating plant growth regulators, increasing osmotic metabolites, and downregulating or upregulating stress-related genes in plants. Recent research studies have also shown that MET treatment increases the stress tolerance of plants. Wei et al. (2018) discovered the receptor of MET, CAND2/PMTR1, in *Arabidopsis*, although the transduction mechanism is not well understood, and whether or not more types of MET receptors exist is unknown. In addition, relatively few research studies have been focused on the genes and core pathways that are specifically regulated by MET. On the other hand, NO is also a signaling molecule involved in numerous physiological functions and therefore plays essential roles in the responses to various abiotic stresses. Some studies have demonstrated that MET is involved in the signaling pathway that is directly mediated by NO, even though their relationship is still unclear. More research studies on endogenous MET and NO are also necessary, as most works have only focused on exogenous MET. Moreover, it is fundamental to understand the different responses depending on the type of stress, and if all of these responses operate through MET and NO. Lastly, S-nitrosation target proteins have not yet been identified, and the NO signaling pathway is not well understood. Ultimately, MET and NO are potentially important molecules in the regulation of ROS and RNS under abiotic stress conditions, but there is still much to understand about how they interact with each other and the nature of their mechanisms of action.

## Figures and Tables

**Table 1:** Selected studies on the roles played by melatonin in the response of plants to abiotic stresses.

Abiotic Stress	Plant Specie	Regulated Element	Reference
Salinity with alcalinity	*Leymus chinensis*	antioxidant systems, photosynthetic activity, arbuscular mycorrhizal fungal growth	[108]
	Tomato (*S. lycopersicum* L.)	photosynthetic activity, lipid peroxidation, capacity of the AsA –GSH cycle, balance of K^+^ and Na^+^	[48]
	Potato (*Ipomoea batatas* L.)	homeostatic balance of Na^+^ and K^+^	[49]
	*Malus hupehensis*	cell membrane damage, root system architecture, SOD, POD, CAT, PAs synthesis	[106]
Salinity	Tomato (*Solanum lycopersicum* L.)	ROS levels, PET, D1 protein.	[136]
	Watermelon (*Citrullus lanatus*)	stomatal closure, light energy absorption, PET in photosystem II, activities of antioxidant enzymes	[107]
	Alfalfa (*Medicago sativa*)	scavenging ROS, activities of antioxidant enzymes	[105]
	(*Fragaria* × *ananassa*)	antioxidant enzymes, ABA	[109]
	Rapeseed (*Brassica napus* L.)	NR and NOA1- dependent NO concentration	[123]
	Sunflower (*Helianthus annuus* L.)	SOD isoforms (Cu/Zn SOD and Mn SOD), NO	[104]
	Sunflower (*H. annuus* L.)	GR (glutathione reductase) activity, GSH content	[47]
	Tomato (*S. lycopersicum* L.)	Na^+^ accumulation, uptake K^+^, antioxidant enzyme activity, AsA–GSH detoxification capacity, NO	[32]
High temperature	Kiwifruit (*Actinidia deliciosa*)	H_2_O_2_ and Pro content, AsA levels, activity of several antioxidant enzymes, GST	[53]
	Tall fescue (*Festuca arundinacea*)	ROS level, EL, membrane lipid peroxidation, MDA, Chl, total protein, antioxidant enzyme activities	[110]
	Maize (*Zea mays* L.)	MDA and EL levels, GR, CAT, AsA, GSH, methylglyoxal detoxification, osmoregulation system	[52]
	Tomato (*S. lycopersicum* L.)	activity of APX and CAT	[50]
	Tomato (*S. lycopersicum* L.)	ROS levels, RuBisCo activity	[143]
	Tomato (*S. lycopersicum* L.)	ROS accumulation in the anthers, activity of antioxidant enzymes, heat shock protein	[111]
	Tomato (*S. lycopersicum* L.)	antioxidant defense mechanisms, AsA-GSH cycle, PAs metabolic pathway, NO	[51]
Low temperature	*Arabidopsis thaliana*	CBFs/DREBs, COR15a, CAMTA1, ZAT10, ZAT12.	[31]
	Cucumber (*Cucumis sativus* L.)	levels of AsA and GSH, SOD, APX, MDHAR, DHAR, GR in the AsA–GSH cycle	[112]
	Cucumber (*C. sativus* L.)	antioxidant enzymes especially SOD and GSSG-R, synthesis of glutathione, GSH/GSSG ratio	[142]
	*Elymus nutans*	ABA, downstream cold-responsive genes such as *EnCBF9*, *EnCBF14*, and *EnCOR14a*	[142]
	Tea (*Camellia sinensis* L.)	photosynthetic capacity, antioxidant potential, redox homeostasis	[95]
	Tomato (*S. lycopersicum* L.)	accumulation of ROS, lipid peroxidation	[36]
	Tomato (*Lycopersicon esculentum*)	arginine pathway activity, PAs, electolyte, MDA, NO accumulation	[145]
Drought	Cucumber (*C. sativus* L.)	photosynthetic rate, Chl degradation, SOD, POD, CAT	[118]
	Naked oat (*Avena nuda* L.)	levels of H_2_O_2_ and O_2_^−.^, SOD, POD, CAT and APX activities, MAPKs, TFs (WRKY1, DREB2 and MYB)	[115]
	Maize (*Z. mays* L.)	photosynthetic efficiency, activities of antioxidants enzymes, soluble proteins, Pro	[113]
	Maize (*Z. mays* L.)	D1 protein, photosynthesic activities, antioxidantive defense system	[116]
	Soybean (*Glycine max* L.	photosystem II efficiency, leaf area index, activity of SOD, POD, CAT, MDA.	[117]
	Chinese hickory (*Carya cathayensis*)	ROS scavenging activity, photosynthetic activity, soluble sugars, Pro	[33]
	*Moringa oleifera* L.	photosynthetic pigments phenolic, antioxidant enzyme systems, MDA content	[98]
	Alfalfa (*Medicago sativa* L.)	SOD, GR, CAT, APX, NR, NadDe	[114]
High light stress	*A. thaliana*	scavenging ROS, antioxidant enzymes	[55]
	*A. thaliana*	photosystems efficiency, oxidative damage	[138]
	*Malus hupehensis*	photosynthetic parameters, Chl fluorescence parameters, stomatal apertures, APX, CAT, POD	[102]
Waterlogging stress	*Malus baccata*	photosynthesis, oxidative damage	[119]
	Alfalfa plants (*Medicago sativa* L.)	ethylene production, PA content	[122]
Fe deficiency	*A. thaliana*	remobilizing cell wall Fe, chlorosis	[144]
N deficiency	Winter wheat (*Triticum aestivum* L.)	N contents and nitrate levels, NR and GS activities	[146]
S deficiency	Tomato (*S. lycopersicum* L.)	ROS content, ChI content, photosynthesis, enzymes involved in S transport and metabolism	[141]
Salinity+high temperatre stress combination	Tomato (*S. lycopersicon*)	antioxidant capacity, photosynthesis parameters, APX, GR, GPX, Ph-GPX	[10]
Low temperature+ droght stress combination	Rice (*Oryza sativa*)	several transporter proteins, Pro content, mitochondrial structure	[18]
High light + N deficiency stress combination	*Haematococcus pluvialis*	astaxanthin, cAMP signaling pathway, signaling cascade of NO-mediated MAPK.	[120]
Fe deficiency + salinity stress combination	Pepper (*Capsicum annuum* L.)	NO, H_2_S	[121]
